# Glycemic Variability and Gut Microbiota Metabolic Patterns: A Novel Perspective on Diabetic Complications

**DOI:** 10.1002/fsn3.71242

**Published:** 2025-12-19

**Authors:** Yongjie Xu, Di Chen, Huiru Yang, Yiqiong Zhang, Changyudong Huang, Yinxue Zhong, Haizhi Li, Liying Zhu, Chengcheng Li, Rui Yan, Mi Liu, Wei Pan

**Affiliations:** ^1^ Prenatal Diagnosis Center Affiliated Hospital of Guizhou Medical University Guiyang Guizhou People's Republic of China; ^2^ School of Basic Medical Sciences Guizhou Medical University Guiyang Guizhou People's Republic of China; ^3^ School of Public Health and Health Management Guizhou Medical University Guiyang Guizhou People's Republic of China; ^4^ Reproductive Medical Center Affiliated Hospital of Guizhou Medical University Guiyang Guizhou People's Republic of China; ^5^ Clinical Laboratory Center Affiliated Hospital of Guizhou Medical University Guiyang Guizhou People's Republic of China; ^6^ Nephrology Department Affiliated Hospital of Guizhou Medical University Guiyang Guizhou People's Republic of China

**Keywords:** diabetic complications, glycemic variability, gut microbiota, metabolic patterns, prevention and therapy

## Abstract

Emerging evidence indicates a critical link between diabetic complications and the interplay of glycemic variability and gut microbiota metabolic activity. This review comprehensively examines the regulatory effects of glycemic fluctuations on the composition and function of the gut microbiota, while elucidating the key mechanisms by which microbial metabolic reprogramming—mediated through short‐chain fatty acids, bile acids, and other metabolites—disrupts host metabolic and immune homeostasis, thereby exacerbating diabetic complications. Research demonstrates that glycemic variability not only directly contributes to vascular and neuronal damage but also remodels the gut microbiota's metabolic network, triggering systemic metabolic dysregulation and chronic low‐grade inflammation. Building on these findings, we explore novel therapeutic strategies targeting the gut microbiota for the prevention and management of diabetic complications. By adopting a “gut‐organ axis” framework, this study unveils the cascade of interactions among glycemic variability, gut microbiota, and metabolic disturbances in the pathogenesis of diabetic complications, offering both theoretical foundations and innovative approaches for clinical intervention.

## Graphical abstract

This review highlights how glycemic variability reshapes gut microbiota composition and function, leading to metabolic reprogramming and the production of key metabolites. These microbial products disrupt host metabolic and immune homeostasis, exacerbating diabetic complications. We propose novel gut microbiota‐targeted therapeutic strategies for mitigating diabetic complications via the gut–organ axis.

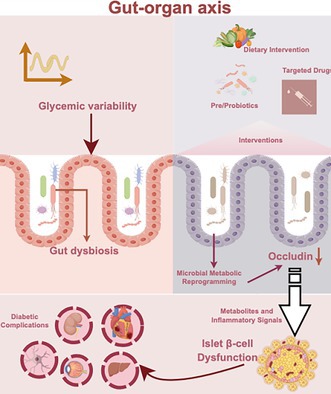

## 1 Introduction

Diabetes mellitus has emerged as one of the most pressing global public health challenges of the 21st century. Recent epidemiological data reveal that the number of individuals with diabetes worldwide has surpassed 537 million, with projections indicating a rise to 783 million by 2045 (Barrea et al. 2025; Moradi Baniasadi et al. 2025). This metabolic disorder not only imposes a substantial disease burden but also contributes to significant morbidity and mortality due to its multisystem complications, including cardiovascular disease, diabetic nephropathy, retinopathy, and peripheral neuropathy (Sedighi et al. 2025). Notably, despite continuous optimization of glycemic control strategies, the incidence of diabetic complications remains persistently high. This clinical conundrum suggests that conventional glycemic markers (e.g., Hemoglobin A1c (HbA1c)) may fail to fully capture the critical pathological mechanisms underlying disease progression. In recent years, growing attention has been directed toward the role of glycemic variability (GV) in diabetic complications (Lucci et al. 2025). Emerging evidence indicates that GV may serve as an independent predictor of microvascular complications, extending beyond its association with long‐term glycemic control (Šoupal et al. 2014). Furthermore, acute hyperglycemia exacerbates tissue damage through metabolic and hemodynamic pathways similar to chronic hyperglycemia (Marcovecchio et al. 2011), reinforcing the limitations of HbA1c as a standalone assessment. Clinically, GV is quantified through parameters including mean glucose levels, standard deviation (SD), and mean amplitude of glycemic excursions (MAGE), which collectively provide a more comprehensive evaluation of glycemic control (Chen, Chen, et al. 2024; Chen, Shen, et al. 2024; Monnier et al. 2025). A study of 34 type 1 diabetes patients revealed significant correlations between glycemic variability indices and metabolic parameters. The average daily risk range and glycemic instability index showed positive correlations with insulin sensitivity (ρ = 0.5 and ρ = 0.48, respectively; *p* < 0.01), while hypoglycemia risk metrics demonstrated a negative correlation with maximal epinephrine response during hypoglycemia (ρ = −0.46, *p* < 0.01) (Pitsillides et al. 2011). These findings underscore the clinical significance of glycemic fluctuation monitoring, which enables timely identification of potential risks and optimization of therapeutic strategies through comprehensive metabolic assessment.

Recent scientific advances have yielded two breakthrough perspectives in understanding diabetic complications. First, technological innovations in continuous glucose monitoring have demonstrated that glycemic variability represents an independent risk factor for complications, distinct from chronic hyperglycemia. Mechanistic studies reveal that glucose fluctuations contribute directly to end organ damage through oxidative stress induction and endothelial dysfunction (Papachristoforou et al. 2020; Klimontov et al. 2021). Emerging evidence has increasingly elucidated the critical interplay between gut microbiota and diabetes pathogenesis. As the largest microbial ecosystem in humans, the intestinal microbiome not only modulates nutrient metabolism and energy homeostasis, but also significantly influences diabetes development through immunomodulatory mechanisms and inflammatory pathway regulation (Tilg and Moschen 2014; Ma et al. 2019). Current research demonstrates a bidirectional relationship between gut dysbiosis and diabetes mellitus, wherein microbial imbalance contributes to disease pathogenesis while diabetic metabolic derangements exacerbate microbiota alterations—a phenomenon termed the “glucose‐microbiota axis” (Li et al. 2018; Wang et al. 2021). Notably, microbial metabolites including short‐chain fatty acids (SCFAs) and bile acids (BAs) play pivotal roles in glycemic regulation through multiple physiological pathways (Takeuchi et al. 2024; Tran et al. 2025). Mechanistic studies reveal that SCFAs enhance hepatic glycogen synthesis by stimulating insulin production through both intestinal G‐protein‐coupled receptor (GPCR) signaling pathways and hepatic AMP‐activated protein kinase (AMPK) activation (Tan et al. 2023; Thiruvengadam et al. 2023). Furthermore, BAs modulate glucose homeostasis by promoting the secretion of Glucagon‐like peptide‐1 (GLP‐1) and peptide YY (PYY) from intestinal L cells and pancreatic α cells (Guo et al. 2024). This bidirectional crosstalk between gut microbiota and diabetes not only provides novel insights into disease pathogenesis, but also suggests potential therapeutic avenues for diabetes prevention and complication management through microbial modulation strategies.

This review presents the first systematic integration of glycemic dynamics and gut microbiota metabolic networks in diabetic complications from a multi‐scale regulatory perspective. By elucidating how microbial metabolic reprogramming amplifies organ damage induced by glycemic fluctuations, we establish a unified theoretical framework for complication pathogenesis while identifying multiple druggable targets. These findings represent a paradigm shift from conventional glycemic control to precision multi‐target intervention strategies, providing both theoretical foundations for microbiome‐based therapeutics and actionable insights for complication prevention.

## 2 Bibliometric Analysis of Glycemic Variability Research: Current Landscape and Trends

### 2.1 Literature Screening and Dataset Construction

**FIGURE 1:**
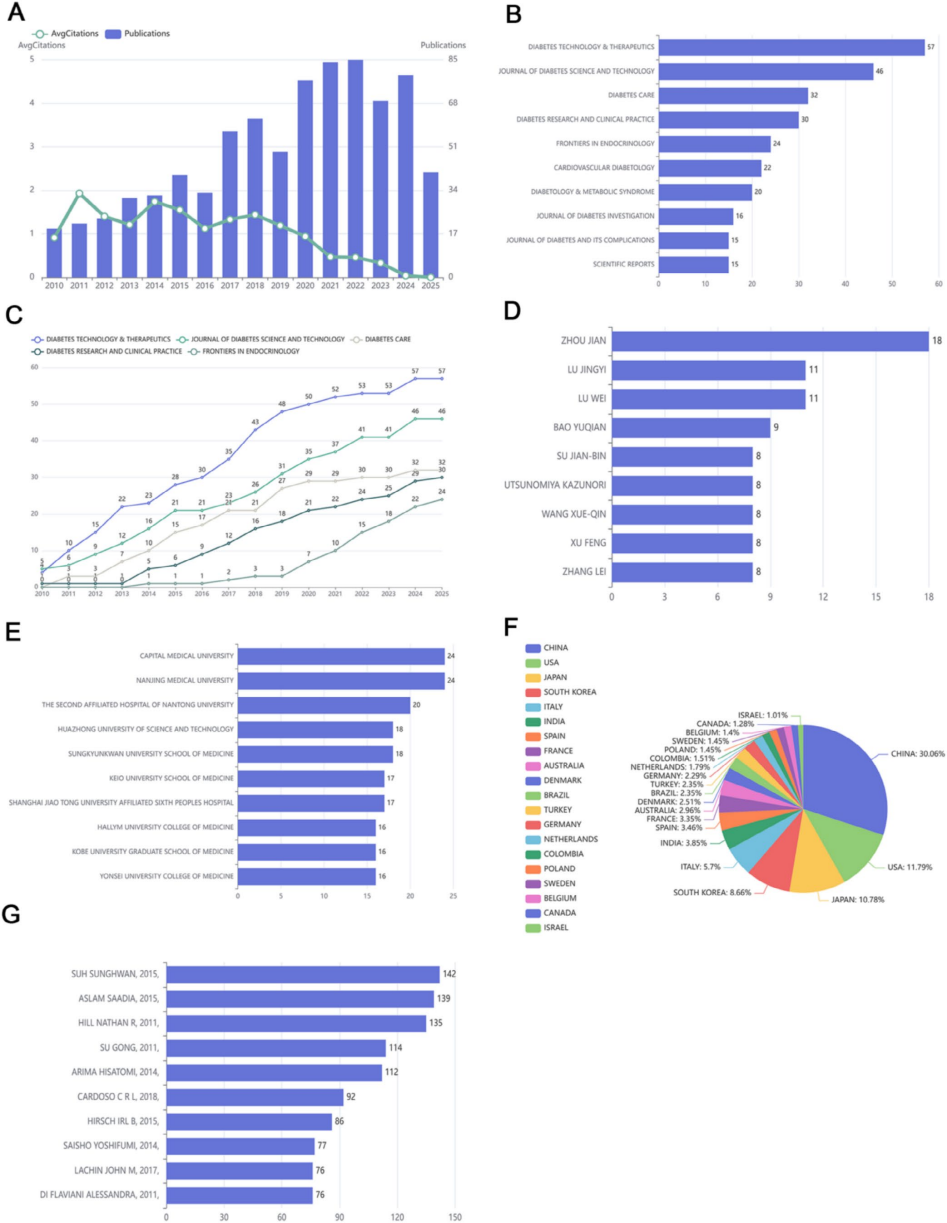
Bibliometric analysis of glycemic variability: (A) Publication trends over the past decade; (B, C) Journals publishing related articles; (D) Authors contributing to the field; (E) Institutions involved in research; (F) Countries with significant contributions; (G) Citation analysis of publications.

Our bibliometric analysis extracted 823 publications from PubMed, including original articles (*n* = 491), reviews (*n* = 58), clinical trials (*n* = 19), case reports (*n* = 2), and other publication types (*n* = 253). The initial dataset spanned publications from 2010 to 2025. After applying a filter to include only publications within the 16‐year period (2010–2025), 802 publications remained eligible for analysis (Figure 1A). Following removal of duplicates and rigorous type‐specific screening, these 802 publications formed the final curated dataset for subsequent analyses.

### 2.2 Publication Trends and Citation Analysis

The annual publication count on glycemic variability research demonstrated consistent growth from 2010 to 2022, peaking in 2021 (*n* = 85) and 2022 (*n* = 89), followed by a modest decline in 2023–2024 (Figure 1A). The mean citation rate per article showed a gradual increase from approximately 1 citation in 2010 to nearly 2 citations by 2018, then entered a downward trajectory approaching zero by 2025 (Figure 1A). This pattern suggests that while research activity has recently tapered, earlier contributions maintain a substantial academic impact in the field.

### 2.3 Analysis of Authors, Institutions, and Journals

A three‐factor analysis revealed collaborative networks among authors, institutions, and journals in glycemic variability research. The top five journals publishing on this topic were *Diabetes Technology & Therapeutics* (*n* = 57), *Journal of Diabetes Science and Technology* (*n* = 46), *Diabetes Care* (*n* = 32), *Diabetes Research and Clinical Practice* (*n* = 30), and *Frontiers in Endocrinology* (*n* = 24), demonstrating their scholarly influence in this field (Figure 1B,C). Key contributors included Fendler Wojciech and Kim Chulho, whose work appeared in high‐impact journals such as *Diabetes Research and Clinical Practice* and *Diabetology & Metabolic Syndrome* (Figure 1C). Su Jian‐bin and Wang Xue‐qin also published significant findings in journals including *Scientific Reports* and *Journal of Diabetes Investigation* (Figure 1C,D). Geographically, China led with 30.06% of publications, followed by the United States (11.79%) and Japan (10.78%). Other active contributors included South Korea (8.66%), Italy (5.70%), and India (3.85%) (Figure 1F). This distribution highlights Asia's prominent role in glycemic variability research, with Western nations following closely. Among the most‐cited works, Suh Sunghwan's 2015 publication ranked first with 142 citations, followed by Aslam Saadia (139 citations, 2015) and Hill Nathan R (135 citations, 2011) (Figure 1G).

### 2.4 Keyword Analysis and Co‐Occurrence Network

**FIGURE 2:**
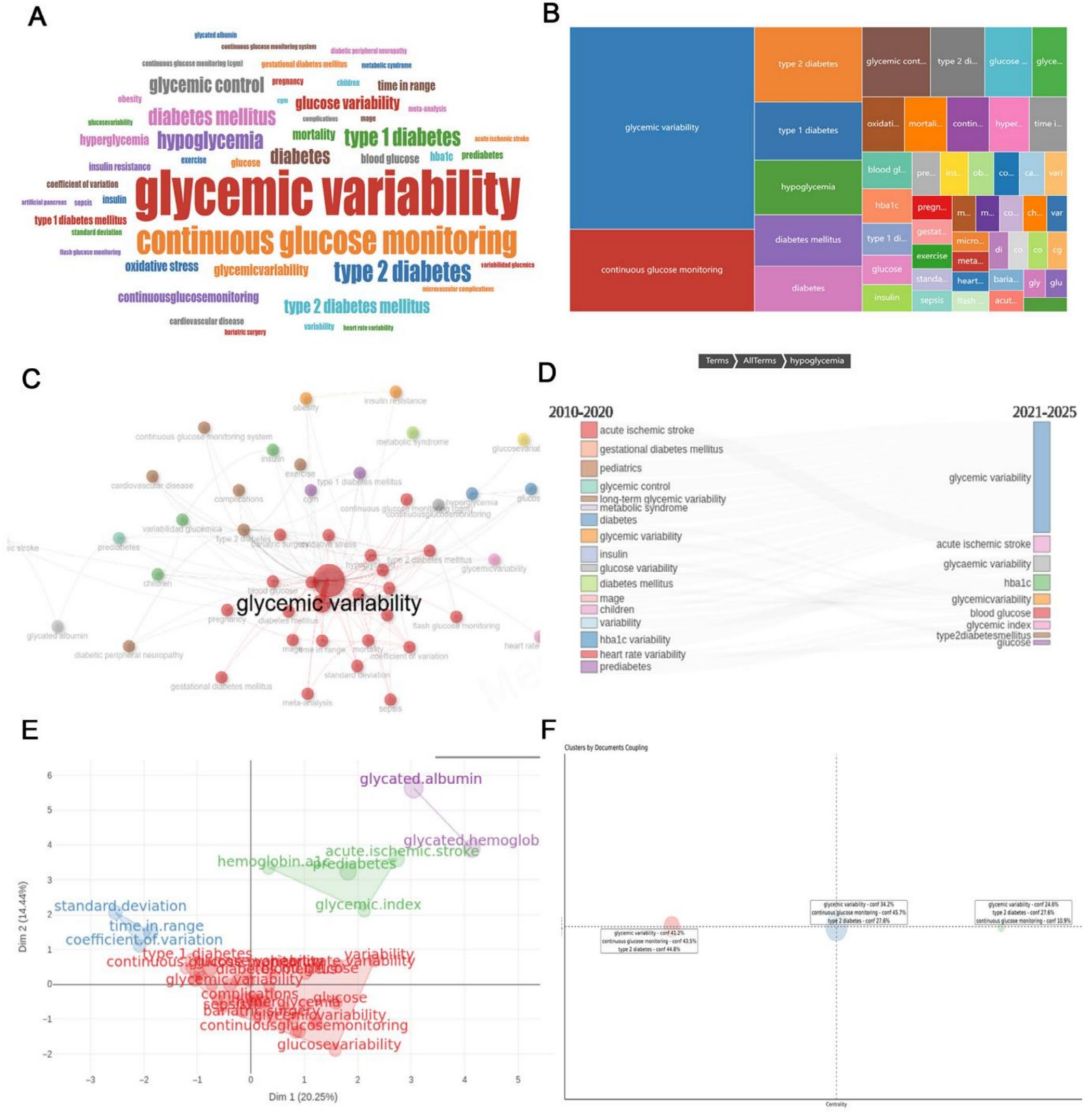
Citation analysis of publications; (A, B) Keyword analysis; (C, D) Topic co‐occurrence network and research hotspot analysis; (E, F) Thematic factor and cluster analysis.

The keyword analysis revealed two predominant research themes: “glycemic variability” and “continuous glucose monitoring” (Figure 2A). High frequency terms including “diabetes mellitus,” “type 1 diabetes,” and “type 2 diabetes” indicated a primary research focus on diabetes classification and management. Additional terms such as “glycemic control,” “hypoglycemia,” and “hyperglycemia” represented key aspects of glucose regulation, while “obesity,” “insulin resistance,” and “cardiovascular disease” highlighted important comorbidities and risk factors (Figure 2B). The co‐occurrence network analysis positioned “glycemic variability” as the central node, demonstrating strong connections with multiple secondary keywords (Figure 2C). Three distinct research clusters emerged from this network: diabetes‐specific investigations (including type 2 diabetes mellitus and gestational diabetes mellitus), glucose monitoring technologies (continuous and flash glucose monitoring systems), and metabolic comorbidities (obesity, insulin resistance, and cardiovascular disease). Temporal analysis revealed an evolution in research focus—while earlier studies (2010–2020) emphasized clinical contexts such as acute ischemic stroke and pediatric populations, recent investigations (2021–2025) have shifted toward more precise metrics including HbA1c and glucose levels while maintaining emphasis on stroke outcomes (Figure 2D). This progression from broad clinical observations to targeted mechanistic studies reflects the field's maturation toward developing more sophisticated management strategies.

### 2.5 Thematic Factor and Cluster Analysis

Thematic factor analysis identified two principal dimensions, accounting for 20.25% and 14.44% of the variance, respectively. The lower‐left quadrant featured keywords such as “standard deviation,” “time in range,” and “type 1 diabetes,” reflecting a focus on glycemic control metrics and management strategies, particularly in type 1 diabetes. In contrast, the upper‐right quadrant included terms like “glycated albumin,” “acute ischemic stroke,” and “hemoglobin A1c,” highlighting long‐term glycemic control and its association with cardiovascular risk (Figure 2E). This suggests that research addresses both short‐term glycemic variability and its long‐term clinical implications. Coupling cluster analysis revealed three distinct clusters. The first cluster (lower‐left) emphasized “glycemic variability,” “continuous glucose monitoring,” and “type 2 diabetes,” with confidence levels of 41.2%, 43.5%, and 44.8%, respectively. A second cluster (central) maintained similar themes but with varying confidence scores (34.2%, 45.7%, and 27.6%), while the third (upper‐right) showed weaker associations (24.6%, 27.6%, and 10.9%) (Figure 2F). These findings indicate consistent research priorities with differing degrees of thematic linkage.

## 3 Physiological and Pathological Mechanisms of Glycemic Variability

### 3.1 Core Regulatory Mechanisms of Glucose Homeostasis

**FIGURE 3:**
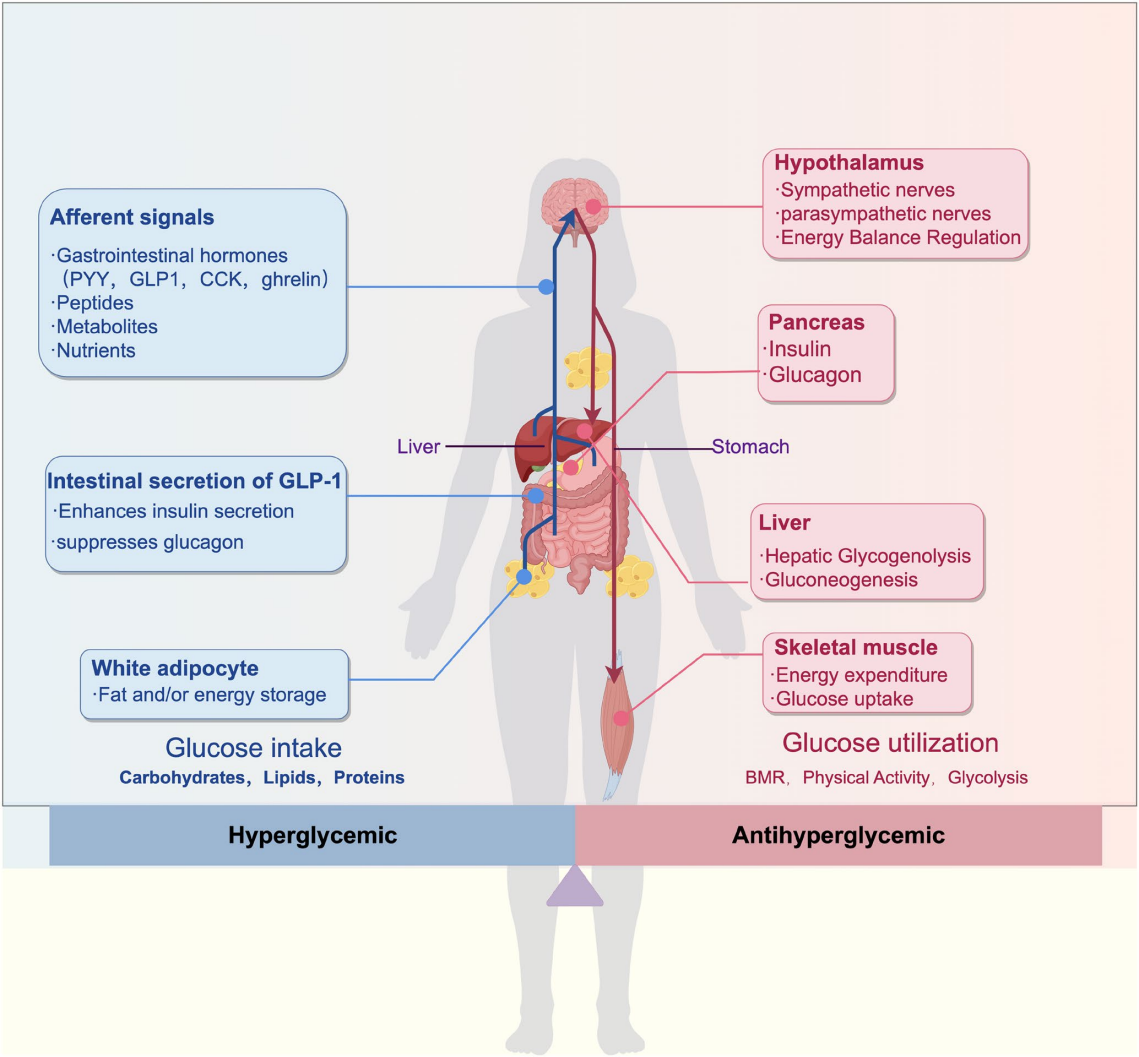
Blood glucose homeostatic regulation mechanism: The diagram uses a human body as the background, labeling various organs and tissues with arrows indicating the pathways of signal transmission and their directions of action.

Glucose homeostasis is maintained through the coordinated interplay of neural, endocrine, and metabolic systems, with pancreatic β‐cell function serving as the central regulator (Henriques et al. 2025) (Figure 3). Under physiological conditions, postprandial hyperglycemia stimulates pulsatile insulin secretion, facilitating glucose uptake in peripheral tissues, while fasting glucose levels are maintained through glucagon‐mediated hepatic glucose output (Kommu et al. 2025; Santos et al. 2025). Aging significantly disrupts this equilibrium: compared to 6‐month‐old rats, 24‐month‐old animals exhibit elevated basal glucose levels during the dark phase (*p* < 0.01), attenuated stress‐induced glycemic responses (42% reduction in peak glucose elevation), and a strong negative correlation between hippocampal synaptic mitochondrial protein expression and diurnal glucose fluctuations (*r* = −0.68) (Braunstein et al. 2024). This dysregulation is closely linked to oxidative stress. In patients with type 2 diabetes, the MAGE shows strong positive correlations with oxidative stress markers, including 8‐isoprostane (*r* = 0.82) and thiobarbituric acid‐reactive substances (*r* = 0.76). Furthermore, glycemic variability directly promotes aortic collagen deposition and vascular dysfunction through activation of the reactive oxygen species (ROS)/p38 mitogen‐activated protein kinase (MAPK)/runt‐related transcription factor 2 (Runx2) signaling pathway (Zhang et al. 2020).

### 3.2 Key Determinants of Glycemic Variability and Their Interactions

Glycemic variability arises from dynamic interactions among metabolic characteristics, hormonal regulation, and lifestyle factors (Canelli et al. 2023; Li et al. 2023). Clinical evidence identifies age, prolonged diabetes duration, and BMI > 31 kg/m^2^ as independent predictors of glycemic instability (Lytrivi et al. 2025; Ortega et al. 2025). In type 1 diabetes, insulin sensitivity positively correlates with MAGE, while impaired epinephrine response during hypoglycemia significantly increases fluctuation risk (Teixeira et al. 2017). Lifestyle interventions demonstrate measurable effects—high glycemic index diets exacerbate postprandial glucose fluctuations in type 2 diabetes, whereas optimized exercise timing enhances glycemic control. Notably, moderate‐intensity exercise initiated 45 min postprandial reduces glucose peaks more effectively than delayed (90‐min) exercise (Qi et al. 2025). These findings provide a mechanistic foundation for personalized glucose management strategies.

### 3.3 Diabetes Subtype‐Specific Glycemic Variability Patterns and Clinical Implications

Type 1 diabetes (T1D) patients exhibit extreme glucose excursions and characteristic “brittle diabetes” patterns due to absolute insulin deficiency and limitations of exogenous insulin replacement therapy, demonstrating significantly greater glycemic variability than type 2 diabetes (T2D) patients (Kuenen, et al. 2011). T2D displays distinct disease‐stage dependent patterns: early‐stage patients primarily show exaggerated postprandial glucose spikes, while late‐stage patients develop increased basal glycemic variability. Large cohort studies confirm that postprandial glucose fluctuations in T2D independently predict cardiovascular event risk (Chen et al. 2010; Lu et al. 2019).

Special diabetes subtypes reveal important mechanistic insights—sulfonylurea‐treated T2D patients demonstrate greater glycemic variability compared to those receiving Dipeptidyl peptidase‐4 (DPP‐4) inhibitors (Yoo et al. 2015). These differences inform both clinical monitoring approaches and personalized treatment strategies, including closed‐loop insulin pump systems for T1D and GLP‐1 receptor agonists (RAs) targeting postprandial fluctuations in T2D (Dandona 2017; Umpierrez and Kovatchev 2018).

### 3.4 Mechanistic Role of Glycemic Variability in Diabetic Complications

**FIGURE 4:**
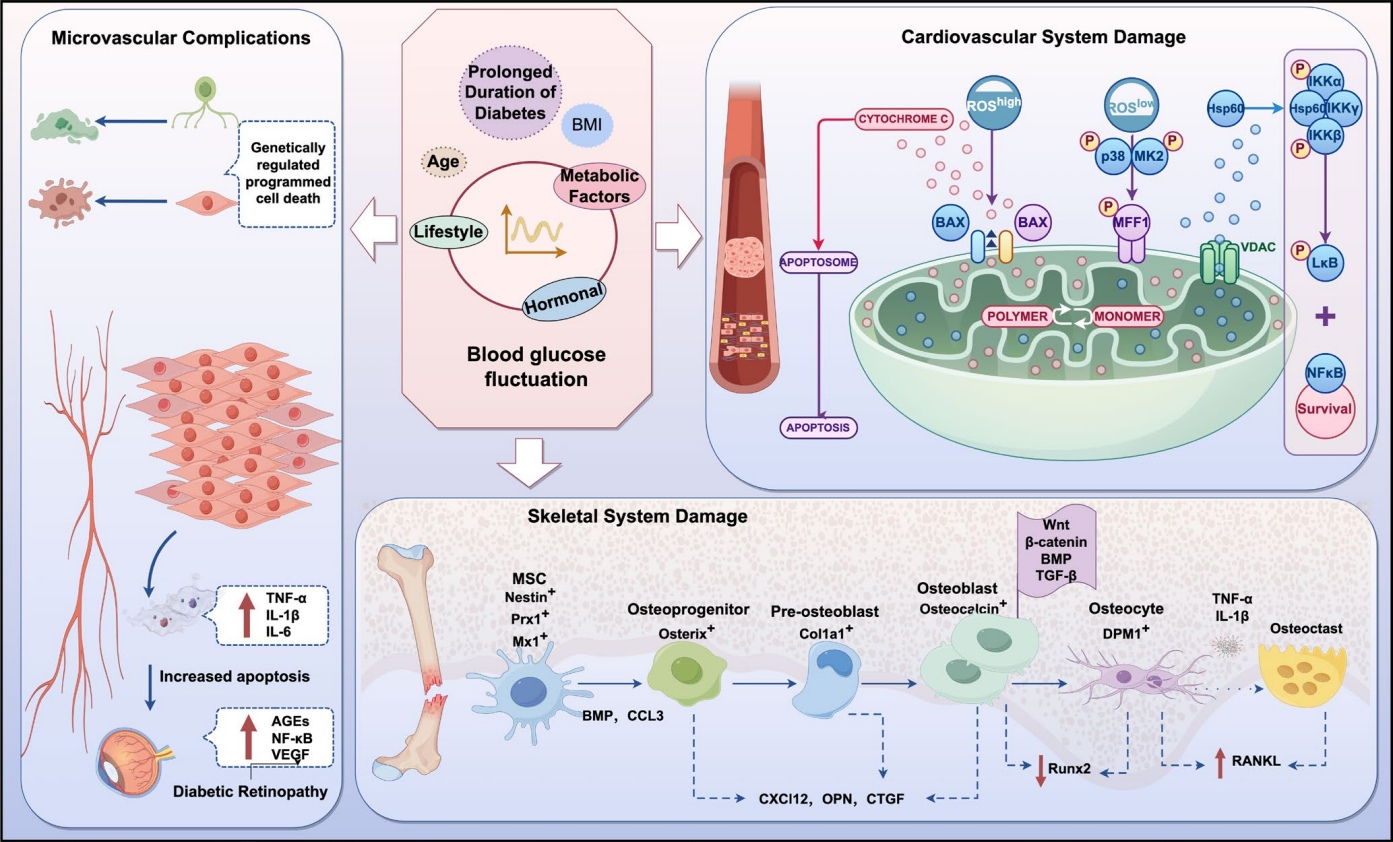
Mechanistic role of glycemic variability in diabetic complications: The diagram is divided into three main sections, describing microvascular complications, cardiovascular system damage, and skeletal system damage.

Glycemic variability has been established as an independent risk factor for diabetic complications through multiple synergistic mechanisms. In the skeletal system, clinical studies demonstrate that greater glucose fluctuations in type 2 diabetes correlate significantly with reduced bone mineral density (β = −0.32, *p* < 0.01) and increased fracture risk (HR = 1.45). This effect is mediated by glucose variability specifically suppressing osteoblast Runx2 expression (58% reduction) while activating osteoclast differentiation signals (Chen, Li, et al. 2025; Chen, Wang, et al. 2025). In cardiovascular complications, animal models reveal that glycemic variability accelerates myocardial fibrosis (2.1‐fold increase in collagen deposition) and diastolic dysfunction (0.35 reduction in E/A ratio) more severely than sustained hyperglycemia. This process is driven by sodium‐glucose cotransporter 1 (SGLT1) ‐mediated mitochondrial ROS overproduction (3.8‐fold increase) (Wu et al. 2021). Epidemiological evidence for microvascular complications shows that each 1 mmol/L increase in MAGE elevates retinopathy progression risk by 18% (95% CI 1.05–1.32), likely due to oscillating hyperglycemia‐induced endothelial apoptosis (2.3‐fold increase) and sustained Interleukin‐6 (IL‐6) secretion (Jung 2015; Klimontov et al. 2021) (Figure 4). Notably, in type 1 diabetes, while the quantitative relationship between glycemic variability and microvascular complications requires larger validation studies, closed‐loop insulin systems that reduce glucose fluctuations improve nerve conduction velocity by 23% (*p* < 0.05), underscoring the therapeutic value of variability control (Smith‐Palmer et al. 2014). These findings collectively establish glycemic variability management as a cornerstone of comprehensive diabetes care and provide a mechanistic basis for targeted interventions (Huang et al. 2023).

## 4 Gut Microbiota: A New Perspective on Blood Glucose Fluctuation Research

### 4.1 Biological Basis of Gut Microbiota Metabolic Patterns

The gut microbiota, a complex microbial community residing in the human intestinal tract, plays a fundamental role in host metabolism through its diverse biochemical transformations (Wen et al. 2022). These microbial populations metabolize dietary components into bioactive compounds that participate in various physiological processes. A prime example is the fermentation of dietary fibers into SCFAs, including acetate, propionate, and butyrate. Beyond serving as energy substrates for colonic epithelial cells, SCFAs modulate glucose metabolism by stimulating intestinal L‐cells to secrete GLP‐1 (Darra et al. 2023). The microbiota also mediates the biotransformation of primary BAs into secondary BAs, which regulate lipid metabolism, energy homeostasis, and intestinal barrier function through specific receptor interactions (Lin et al. 2023). Notably, microbial‐derived trimethylamine N‐oxide (TMAO) has emerged as a risk factor for cardiovascular diseases, highlighting the systemic impact of gut microbial metabolism (Wen et al. 2022; Shuai et al. 2025). Furthermore, bacterial processing of tryptophan yields various indole derivatives that participate in immune regulation and inflammatory responses within the gut microenvironment (Miao et al. 2025; Zhao et al. 2025).

### 4.2 Gut Microbiota Metabolic Patterns in Diabetic Complications

**FIGURE 5:**
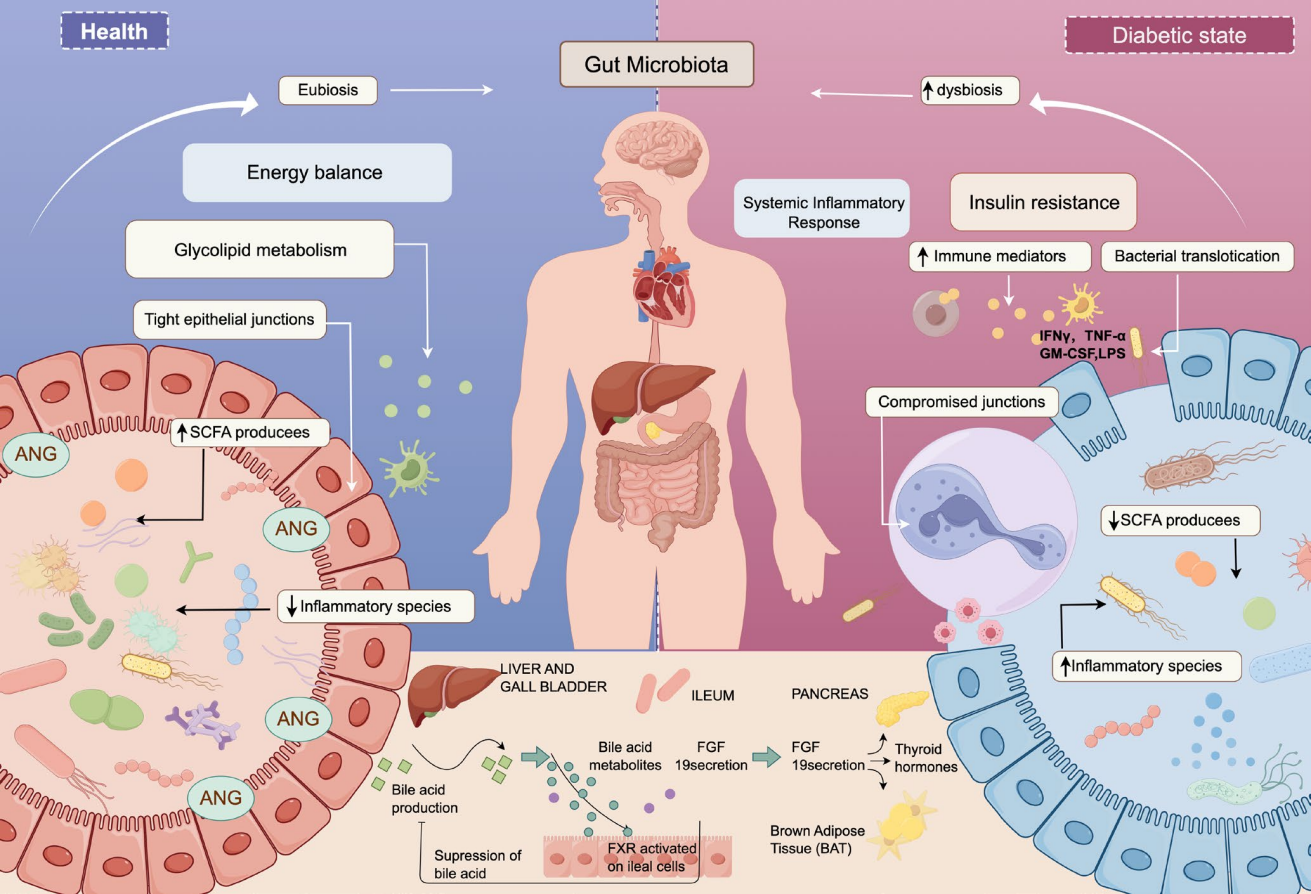
Metabolic patterns of intestinal flora in normal and diabetic states: The left side describes the normal gut microbiota metabolic pattern, while the right side describes the state of diabetes, where gut microbiota dysbiosis is a key factor in the pathogenesis of diabetic complications. The lower part of the image illustrates BA metabolism and its impact on multiple organ systems.

Growing evidence implicates gut microbiota dysbiosis as a critical contributor to the pathogenesis of diabetic complications. Patients with diabetes exhibit distinct gut microbial profiles compared to healthy individuals, which may promote intestinal barrier dysfunction, chronic low‐grade inflammation, and metabolic endotoxemia (van Olden et al. 2015; Guo et al. 2022). The gut microbiota influences diabetic complications through several interconnected mechanisms. Microbial dysbiosis compromises intestinal barrier integrity, increasing permeability to bacterial endotoxins such as lipopolysaccharide. This triggers systemic inflammation that exacerbates insulin resistance and tissue damage (Sohail et al. 2017; Guo et al. 2022). Microbial metabolites, particularly SCFAs and BAs, play direct roles in metabolic regulation (Parada Venegas et al. 2019; Wang et al. 2019). SCFAs enhance insulin sensitivity and modulate enteroendocrine hormone secretion to improve glucose and lipid homeostasis (Crommen and Simon 2017; Spiljar et al. 2017), while BAs regulate energy metabolism and inflammatory responses through receptor‐mediated signaling (Liu, Jin, et al. 2024; Liu, Zhang, et al. 2024; Xu et al. 2024). Furthermore, gut microbiota alterations disrupt immune homeostasis, leading to aberrant immune activation that amplifies inflammatory tissue injury (Alvarez‐Vieites et al. 2020; Chen, Chen, et al. 2024; Chen, Shen, et al. 2024). Targeting microbial composition may represent a therapeutic strategy to restore immune balance and alleviate complications (Udayappan et al. 2014; Lundgrin and Hatipoglu 2025) (Figure 5).

As discussed previously, glycemic variability significantly contributes to diabetic complications. An important unanswered question is whether glucose fluctuations reciprocally alter gut microbial metabolism, thereby creating a vicious cycle that accelerates disease progression. This potential bidirectional relationship merits further investigation.

### 4.3 Bidirectional Interaction Between Glycemic Variability and Gut Microbiota Metabolism

Emerging evidence reveals a complex reciprocal relationship between glycemic fluctuations and gut microbial ecology. Clinical and preclinical studies demonstrate that oscillating glucose levels significantly alter gut microbiota composition and functionality. Hyperglycemic conditions modify intestinal microenvironmental parameters including pH and redox potential, creating selective pressures that reshape microbial communities (Darra et al. 2023; Zong et al. 2024). In diabetic murine models, glycemic variability correlates with reduced microbial diversity, characterized by depletion of beneficial taxa (e.g., *Bifidobacterium, Lactobacillus*) and expansion of potentially pathogenic Enterobacteriaceae (Guo et al. 2024). Conversely, gut microbiota and their metabolic byproducts actively modulate glycemic responses. Microbial‐derived SCFAs stimulate G protein‐coupled receptors (GPCRs) on enteroendocrine cells, enhancing GLP‐1 secretion to promote insulin release and glucose homeostasis (Zhang et al. 2019). Dietary fibers like pectin undergo microbial fermentation to yield SCFAs and secondary BAs, which coordinate glucose regulation through multiple pathways (Elshahed et al. 2021; Yin et al. 2024). Beyond direct metabolic regulation, gut microbiota indirectly influence glycemic variability through modulation of intestinal barrier integrity, systemic inflammation, and immune homeostasis (Jiang et al. 2025; Zheng et al. 2025). These findings collectively suggest that glycemic fluctuations and gut microbial metabolism engage in continuous crosstalk, potentially establishing a vicious cycle in diabetes progression. Further investigation is warranted to elucidate the temporal dynamics and therapeutic implications of this bidirectional relationship.

**FIGURE 6:**
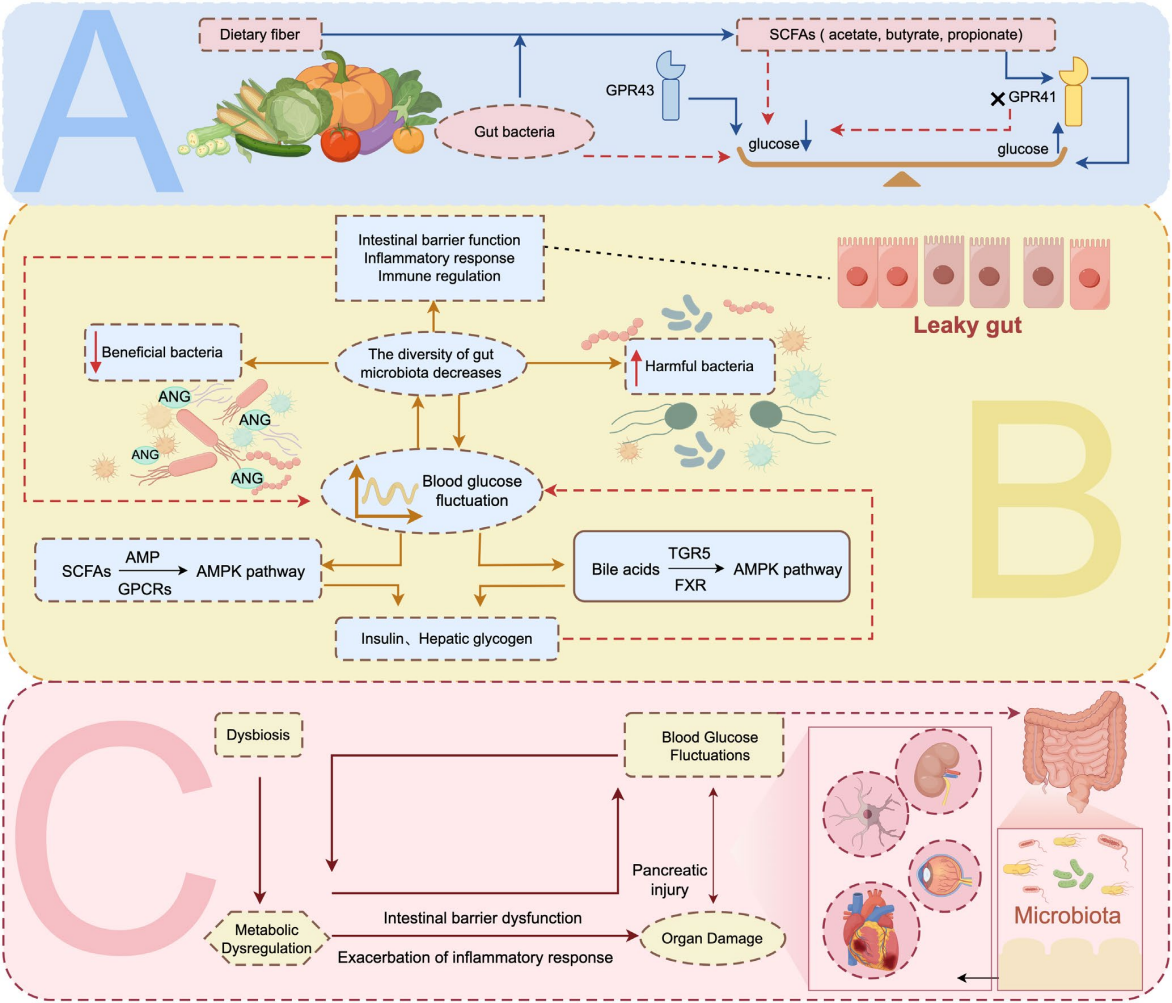
Bidirectional interaction between glycemic variability and gut microbiota metabolism: (A) Dietary fiber is fermented by gut bacteria to produce SCFAs like acetate, butyrate, and propionate. G protein‐coupled receptor 43 (GPR43) receptors lower blood glucose, while G protein‐coupled receptor 41 (GPR41) may raise it; (B) Blood glucose fluctuations affect the stability of gut microbiota and BA metabolism, leading to imbalances in intestinal barrier integrity, systemic inflammation, and immune homeostasis, which indirectly influence blood glucose fluctuations; (C) Glucose dysregulation‐microbial dysbiosis‐metabolic dysfunction‐end organ damage.

These findings reveal a sophisticated bidirectional regulatory network between glycemic variability and gut microbiota, where metabolic perturbations may establish a self‐perpetuating cycle of “glucose dysregulation‐microbial dysbiosis‐metabolic dysfunction‐end organ damage.” This vicious cycle likely represents a fundamental mechanism driving the progression of diabetic complications, offering novel insights into disease pathogenesis. While this association remains underexplored in diabetes research, the underlying mechanisms may provide explanatory power for key pathological features including insulin resistance and chronic inflammation—hallmarks of diabetic complications. Our synthesis of current evidence positions gut microbial metabolic patterns as central players in complication development, moving beyond the conventional glucocentric paradigm. Notably, microbial metabolites (e.g., butyrate, secondary BAs) demonstrate dual functionality: directly modulating host metabolism while potentially influencing target organ function through epigenetic regulation. Future investigations should prioritize exploring microbial metabolic interventions (e.g., targeted probiotics, dietary fibers) for their potential to simultaneously ameliorate glycemic variability and prevent complications. Such approaches could inform precision medicine strategies for diabetes management. Elucidation of these mechanisms promises to open new avenues for both early prevention and targeted therapy of diabetic complications, potentially revolutionizing clinical approaches to this pervasive metabolic disorder (Figure 6).

## 5 Therapeutic Approaches for Glycemic Variability and Gut Microbiota Dysregulation

### 5.1 Pharmacological and Non‐Pharmacological Strategies for Glycemic Control

Effective management of blood glucose fluctuations is a critical intervention for preventing diabetes complications, with existing approaches encompassing both pharmacological and non‐pharmacological therapies (Kovatchev 2012). The efficacy of various antidiabetic medications in regulating glucose fluctuations varies significantly. DPP‐4 inhibitors, exemplified by vildagliptin, reduce postprandial glucose fluctuations by enhancing endogenous GLP‐1 activity (Chen et al. 2019). A comparative study involving 24 type 2 diabetes patients demonstrated that vildagliptin could reduce mean A1c equivalent (MAGE) by approximately 20% compared to glibenclamide. It also significantly elevated active GLP‐1 levels and inhibited glucagon secretion, though the MAGE reduction was not statistically significant (He et al. 2013). The combination therapy of glucagon‐like peptide‐1 (GLP‐1) RAs with basal insulin provides a more effective glucose management strategy for type 2 diabetes patients, particularly suitable for those requiring stable glucose fluctuations (Lin et al. 2022). SGLT2 inhibitors (SGLT2i) increase urinary glucose excretion and lower blood glucose levels by inhibiting the SGLT2 transporter in the proximal renal tubule without insulin dependence (Sakai et al. 2025). These medications demonstrate dual therapeutic advantages through promoting glucose excretion (glycosuria) and improving glucose fluctuations (Zhang et al. 2025). Currently, combination therapy with GV and metformin is more effective than monotherapy for patients undergoing lifestyle interventions or those already using metformin, without inducing hypoglycemia (Park et al. 2022). GV is associated with increased mortality in critically ill patients. Even in cases of mild hyperglycemia, clinical outcomes with smaller glucose fluctuations are superior to those achieved through strict glycemic control with larger fluctuations. Insulin infusion remains the gold standard for managing stress‐induced hyperglycemia in critically ill patients. Optimized insulin therapy, particularly through continuous subcutaneous insulin infusion (CSII) compared to multiple daily injections, demonstrates superior glycemic stability. A study involving 80 critically ill patients revealed that insulin infusion therapy (IIT) significantly reduced GV. Using the Continuous Overlapping Net Glycemic Action (CONGA) metric, which measures glycemic variability over time, GV decreased by 0.65 (95% CI [−1.16, −0.14], *p* = 0.01) without increasing the incidence of severe hypoglycemia. Compared to the insulin sliding scale (ISS), the IIT group required correction for hypoglycemia at a rate of 2.77% (*p* = 0.38), down from 6.8%. Further research indicates that IIT shows significant advantages in reducing glycemic variability, though its impact on improving survival rates in critically ill patients requires validation through larger‐scale studies (Almagthali et al. 2024).

Non‐pharmacological approaches center on dietary modification and exercise interventions. Strategic carbohydrate management coupled with increased dietary fiber intake effectively mitigates glucose fluctuations. Exercise regimens incorporating combined aerobic and resistance training show measurable impact. This was demonstrated by a study of 50 type 2 diabetes patients showing significant reductions in sensor glucose standard deviation (SDSG) and CV after 1 week of supervised training (Liu, Jin, et al. 2024; Liu, Zhang, et al. 2024). A study involving 252 participants found that regular physical activity (PA) and adherence to the Mediterranean diet (MeDi) independently improved glycemic control and clinical outcomes in patients with T2DM (Radić et al. 2025).

### 5.2 Microbiota‐Targeted Interventions in Diabetes Management

Emerging evidence highlights gut microbiota modulation as a promising therapeutic strategy for diabetes, with current approaches encompassing dietary modifications, probiotic/prebiotic supplementation, and fecal microbiota transplantation (FMT) (Fernández‐Ruiz 2021; Chen, Li, et al. 2025; Chen, Wang, et al. 2025). Dietary intervention represents a fundamental approach, with fiber‐rich diets demonstrating particular efficacy in promoting beneficial bacterial growth and enhancing SCFA production (Akanyibah et al. 2025; Zhou et al. 2025). Clinical observations confirm that oat bran, inulin, and other fiber‐rich substrates significantly alter microbial metabolic patterns, increasing SCFA generation and consequently improving glycemic control (Dell'Olio et al. 2024). Probiotic administration directly modifies gut microbial composition, with studies demonstrating that *Bifidobacterium* supplementation enhances intestinal barrier function, reduces inflammatory markers, and improves glucose metabolism in diabetic models (Guo et al. 2024). Prebiotics such as fructooligosaccharides selectively stimulate beneficial bacterial proliferation, with documented effects on ameliorating insulin resistance through microbial ecosystem modulation (Zhang et al. 2022). While FMT shows therapeutic potential in restoring microbial homeostasis for diabetes management, its clinical application remains constrained by technical challenges and safety considerations (Su et al. 2025; Yan et al. 2025). This intervention requires further standardization and rigorous evaluation before widespread implementation (Kraithong et al. 2025).

### 5.3 Integrated Therapeutic Approaches for Diabetes and Its Complications

Emerging evidence underscores the critical role of glycemic variability in diabetes progression through its modulation of gut microbiota metabolic reprogramming. This mechanistic insight necessitates a paradigm shift in treatment strategies, where optimal glycemic control must be coupled with microbiota stabilization for maximal therapeutic efficacy (Ebrahimi et al. 2024). Pharmacologically, combination therapies leveraging complementary mechanisms demonstrate synergistic benefits (Nyambuya et al. 2020; Ma et al. 2024). GLP‐1 RAs exemplify this dual action, exhibiting not only glucose‐lowering effects but also microbiota‐modulating properties via intestinal hormone regulation and barrier function enhancement (Chu et al. 2025; Verma et al. 2025). Clinical data reveal significant microbial composition changes in type 2 diabetes patients following 48‐week dulaglutide treatment, suggesting microbiota‐mediated glycemic benefits (Liang et al. 2024). Metformin represents another multifaceted agent, with proven capabilities in both glycemic control and microbiota modulation. Beyond its primary antidiabetic action, metformin increases beneficial bacterial abundance, thereby improving gut microbial ecology (Sun et al. 2018). These findings highlight the therapeutic advantage of agents that simultaneously target glycemic stability and microbial homeostasis.

FMT demonstrates considerable therapeutic potential, with preclinical studies confirming its efficacy in restoring microbial diversity and ameliorating metabolic endotoxemia in diabetic models (Suez et al. 2018; Zikou et al. 2024; Qin, Fan, et al. 2025; Qin, Zheng, et al. 2025). Multi‐omics analyses integrating 16S rRNA sequencing, metagenomics, and metabolomics reveal substantial interindividual variability in gut microbial composition and metabolic patterns among diabetes patients (Peng et al. 2019; Qin, Fan, et al. 2025; Qin, Zheng, et al. 2025), providing a scientific foundation for personalized intervention strategies. We propose a novel precision probiotic therapy framework targeting specific microbial consortia involved in SCFA production and BA metabolism. This approach achieves dual improvement in glycemic variability and systemic inflammation by strategically modulating microbial networks. Such targeted interventions may disrupt the vicious cycle of “hyperglycemia‐dysbiosis‐metabolic dysfunction,” representing a paradigm shift in diabetes therapeutics. The development of predictive microbial‐metabolic signatures will be crucial for standardizing personalized microbiota‐based interventions. Future research should focus on establishing response biomarkers to optimize both glycemic control and complication prevention, while maintaining cost‐effectiveness in clinical implementation.

## 6 Outlook

### 6.1 Development of Personalized Glycemic Variability Monitoring

Technological innovations and personalized medicine approaches are converging to transform glycemic control strategies. Recent advances in noninvasive glucose monitoring have yielded novel wearable devices incorporating microneedle arrays and wireless transmission, demonstrating both high accuracy and biocompatibility (Tong et al. 2025). Concurrently, artificial intelligence has revolutionized glucose management, with reinforcement learning algorithms optimizing insulin regimens by analyzing large‐scale glycemic data to improve time‐in‐range (TIR) metrics (Dénes‐Fazakas et al. 2024). Precision medicine applications now enable genotype‐guided pharmacotherapy, particularly in monogenic diabetes where genetic profiling directs optimal drug selection (Elk and Iwuchukwu 2017; Foddha et al. 2025). Lifestyle interventions have similarly evolved toward personalization, with body composition analysis (including visceral adipose tissue and mid‐thigh muscle area) informing targeted exercise protocols that more effectively mitigate glycemic excursions in type 2 diabetes (Liu, Jin, et al. 2024; Liu, Zhang, et al. 2024). The integration of these technological and clinical advances provides a multidimensional framework for precision glycemic management, ushering in an era of intelligent, individualized diabetes care.

### 6.2 Glycemic Variability and Gut Microbiota: Research Challenges and Future Directions

The causal relationship between glycemic variability and gut microbiota remains contentious, with bidirectional mechanisms incompletely understood. Current evidence suggests hyperglycemia alters the intestinal microenvironment (e.g., osmotic pressure, inflammation), suppressing beneficial bacteria (e.g., *Bifidobacterium*, *Lactobacillus*) while promoting pathogenic colonization (e.g., Enterobacteriaceae), thereby compromising gut barrier function and exacerbating metabolic dysregulation. Conversely, emerging hypotheses posit that microbial dysbiosis may initiate glycemic fluctuations via endotoxemia, chronic low‐grade inflammation, and insulin resistance. Key limitations include reliance on cross‐sectional data and animal models, which obscure temporal causality, alongside confounding variables like host genetics and dietary interventions. Future studies should employ longitudinal cohorts, interventional trials (e.g., microbiota transplantation or targeted glycemic control), and causal inference methods (e.g., Mendelian randomization) to delineate directional relationships and molecular interaction networks.

### 6.3 Future Directions in Glycemic Variability and Gut Microbiota Research

Advancements in multi‐omics technologies and dynamic monitoring systems are driving three fundamental paradigm shifts in this field: First, *dynamic interaction mapping* will emerge through synchronized continuous glucose monitoring (CGM) and real‐time microbial metabolite profiling (e.g., SCFAs, secondary BAs), enabling spatiotemporal resolution of host‐microbiome crosstalk. Second, *precision modulation strategies* will evolve by leveraging host‐microbial co‐metabolic signatures (e.g., personalized metagenomic and metabolic phenotypes) to develop targeted probiotic/prebiotic formulations, bacteriophage therapies, and chrono‐optimized antidiabetic drug regimens. Third, *convergence science approaches* will integrate organoid models, gut‐on‐a‐chip microfluidics, and machine learning‐derived biomarker networks from high‐dimensional datasets, facilitating diabetes endotyping and novel therapeutic targeting.

These transformative approaches promise to resolve existing mechanistic controversies while accelerating clinical translation of “gut‐glycemic axis” interventions—shifting the therapeutic paradigm from symptomatic management to etiology‐targeted treatment.

## 7 Conclusion

Through systematic review and in‐depth analysis, this study reveals the complex interaction between blood glucose fluctuations and gut microbiota metabolic activities in the development of diabetes complications. The research demonstrates that blood glucose fluctuations not only directly induce vascular and neural damage, but also trigger systemic metabolic disorders and chronic inflammation by reshaping the metabolic network of gut microbiota, thereby accelerating the progression of diabetes complications. Based on the “gut‐organ axis” theoretical framework, this paper systematically elucidates the cascade mechanism linking blood glucose fluctuations, gut microbiota dysregulation, metabolic disorders, and organ damage. These findings provide new theoretical support and potential therapeutic pathways for the prevention and clinical management of diabetes complications. Future research should focus on developing personalized blood glucose monitoring technologies, conducting in‐depth analysis of molecular mechanisms underlying blood glucose fluctuations–gut microbiota interactions, and designing targeted comprehensive intervention strategies to offer more precise and effective treatment options for diabetes patients.

## Author Contributions

Yongjie Xu, Di Chen, and Wei Pan contributed to the study's conceptualization, methodology, and original draft preparation; Huiru Yang, Yiqiong Zhang, and Mi Liu were responsible for data curation, formal analysis, and visualization; Changyudong Huang, Yinxue Zhong, and Haizhi Li conducted the investigation and provided resources; Liying Zhu and Chengcheng Li performed validation and reviewed and edited the manuscript; Rui Yan supervised the project, administered its execution, and acquired funding. All authors read and approved the final manuscript. Yongjie Xu is the guarantor of this work and, as such, had full access to all the data in the study and takes responsibility for the integrity of the data and the accuracy of the data analysis.

## Funding

This research was funded by the following grants: National Natural Science Foundation of China (Grant Nos. 82260165, 82300920, 82560169), Cultivation Project for the National Natural Science Foundation of China, Affiliated Hospital of Guizhou Medical University (Grant Nos. 2021‐10, 2022‐37), Guizhou Provincial Science and Technology Plan Project (Grants: Qian Ke He Foundation‐ZK[2024] General 199, Qian Ke He Support‐[2025] General 120), Scientific and Technological Fund of Guizhou Provincial Health Commission (Grant No. gzwkj2024‐081).

## Ethics Statement

As this is a review article, no ethical approval was required, as the study did not involve human or animal participants.

## Consent

All authors have provided their explicit consent for the publication of this manuscript.

## Conflicts of Interest

The authors declare no conflicts of interest.

## Data Availability

Data sharing not applicable to this article as no datasets were generated or analyzed during the current study.

## References

[fsn371242-bib-0001] Akanyibah, F. A. , C. He , X. Wang , B. Wang , and F. Mao . 2025. “The Role of Plant‐Based Dietary Compounds in Gut Microbiota Modulation in Inflammatory Bowel Disease.” Frontiers in Nutrition 12: 1606289. 40521353 10.3389/fnut.2025.1606289 PMC12163340

[fsn371242-bib-0002] Almagthali, A. , S. Alsohimi , A. Alkhalaf , K. A. Sulaiman , and O. Aljuhani . 2024. “Assessing Glycemic Variability in Critically Ill Patients: A Prospective Cohort Study Comparing Insulin Infusion Therapy With Insulin Sliding Scale.” Scientific Reports 14, no. 1: 10128. 38698018 10.1038/s41598-024-57403-5 PMC11066101

[fsn371242-bib-0003] Alvarez‐Vieites, E. , A. López‐Santamarina , J. M. Miranda , et al. 2020. “Influence of the Intestinal Microbiota on Diabetes Management.” Current Pharmaceutical Biotechnology 21, no. 15: 1603–1615. 32410561 10.2174/1389201021666200514220950

[fsn371242-bib-0004] Barrea, L. , L. Verde , A. Colao , L. J. Mandarino , and G. Muscogiuri . 2025. “Medical Nutrition Therapy for the Management of Type 2 Diabetes Mellitus.” Nature Reviews. Endocrinology 21: 769–782. 10.1038/s41574-025-01161-5 40817355

[fsn371242-bib-0005] Braunstein, P. W. , D. J. Horovitz , A. M. Hampton , et al. 2024. “Daily Fluctuations in Blood Glucose With Normal Aging Are Inversely Related to Hippocampal Synaptic Mitochondrial Proteins.” Aging Brain 5: 100116. 38596458 10.1016/j.nbas.2024.100116 PMC11002859

[fsn371242-bib-0006] Canelli, R. , J. Louca , C. Hartman , and F. Bilotta . 2023. “Preoperative Carbohydrate Load to Reduce Perioperative Glycemic Variability and Improve Surgical Outcomes: A Scoping Review.” World Journal of Diabetes 14, no. 6: 783–794. 37383597 10.4239/wjd.v14.i6.783 PMC10294067

[fsn371242-bib-0007] Chen, B. , C. Shen , and B. Sun . 2024. “Current Landscape and Comprehensive Management of Glycemic Variability in Diabetic Retinopathy.” Journal of Translational Medicine 22, no. 1: 700. 39075573 10.1186/s12967-024-05516-w PMC11287919

[fsn371242-bib-0008] Chen, C. , Y. Huang , Y. Zeng , X. Lu , and G. Dong . 2019. “Targeting the DPP‐4‐GLP‐1 Pathway Improves Exercise Tolerance in Heart Failure Patients: A Systematic Review and Meta‐Analysis.” BMC Cardiovascular Disorders 19, no. 1: 311. 31870322 10.1186/s12872-019-01275-5 PMC6927173

[fsn371242-bib-0009] Chen, F. , P. Wang , F. Dai , et al. 2025. “Correlation Between Blood Glucose Fluctuations and Osteoporosis in Type 2 Diabetes Mellitus.” International Journal of Endocrinology 2025: 8889420. 39949568 10.1155/ije/8889420 PMC11824305

[fsn371242-bib-0010] Chen, L. , Q. Chen , S. Chao , Z. Yuan , L. Jia , and Y. Niu . 2024. “Influence of Gut Flora on Diabetes Management After Kidney Transplantation.” BMC Nephrology 25, no. 1: 468. 39716100 10.1186/s12882-024-03899-y PMC11665093

[fsn371242-bib-0011] Chen, L. , B. Li , M. Zu , R. L. Reis , S. C. Kundu , and B. Xiao . 2025. “Advances and Mechanisms of Gut Microbiota Modulation in Enhancing Immune Checkpoint Inhibitor Efficacy.” Seminars in Cancer Biology 114: 150–172. 40617533 10.1016/j.semcancer.2025.06.012

[fsn371242-bib-0012] Chen, X. , Y. Zhang , X. Shen , et al. 2010. “Correlation Between Glucose Fluctuations and Carotid Intima‐Media Thickness in Type 2 Diabetes.” Diabetes Research and Clinical Practice 90, no. 1: 95–99. 20605247 10.1016/j.diabres.2010.05.004

[fsn371242-bib-0013] Chu, N. , J. Ling , E. Poon , et al. 2025. “Combining a Diet Rich in Fermentable Carbohydrates With Metformin Improves Glycaemic Control and Reshapes the Gut Microbiota in People With Prediabetes.” Nature Metabolism 7, no. 8: 1614–1629. 10.1038/s42255-025-01336-4 40745466

[fsn371242-bib-0014] Crommen, S. , and M. Simon . 2017. “Microbial Regulation of Glucose Metabolism and Insulin Resistance.” Genes 9, no. 1: 10. 29286343 10.3390/genes9010010 PMC5793163

[fsn371242-bib-0015] Dandona, P. 2017. “Minimizing Glycemic Fluctuations in Patients With Type 2 Diabetes: Approaches and Importance.” Diabetes Technology & Therapeutics 19, no. 9: 498–506. 28771387 10.1089/dia.2016.0372 PMC5647495

[fsn371242-bib-0016] Darra, A. , V. Singh , A. Jena , et al. 2023. “Hyperglycemia Is Associated With Duodenal Dysbiosis and Altered Duodenal Microenvironment.” Scientific Reports 13, no. 1: 11038. 37419941 10.1038/s41598-023-37720-x PMC10329043

[fsn371242-bib-0017] Dell'Olio, A. , J. Rubert , V. Capozzi , et al. 2024. “Non‐Invasive VOCs Detection to Monitor the Gut Microbiota Metabolism In‐Vitro.” Scientific Reports 14, no. 1: 15842. 38982163 10.1038/s41598-024-66303-7 PMC11233675

[fsn371242-bib-0018] Dénes‐Fazakas, L. , L. Szilágyi , L. Kovács , A. De Gaetano , and G. Eigner . 2024. “Reinforcement Learning: A Paradigm Shift in Personalized Blood Glucose Management for Diabetes.” Biomedicine 12, no. 9: 2143. 10.3390/biomedicines12092143 PMC11429314 39335656

[fsn371242-bib-0019] Ebrahimi, M. , H. Ahmadieh , M. R. Kanavi , et al. 2024. “Shared Signaling Pathways and Comprehensive Therapeutic Approaches Among Diabetes Complications.” Frontiers in Medicine 11: 1497750. 39845838 10.3389/fmed.2024.1497750 PMC11750824

[fsn371242-bib-0020] Elk, N. , and O. F. Iwuchukwu . 2017. “Using Personalized Medicine in the Management of Diabetes Mellitus.” Pharmacotherapy 37, no. 9: 1131–1149. 28654165 10.1002/phar.1976

[fsn371242-bib-0021] Elshahed, M. S. , A. Miron , A. C. Aprotosoaie , and M. A. Farag . 2021. “Pectin in Diet: Interactions With the Human Microbiome, Role in Gut Homeostasis, and Nutrient‐Drug Interactions.” Carbohydrate Polymers 255: 117388. 33436217 10.1016/j.carbpol.2020.117388

[fsn371242-bib-0022] Fernández‐Ruiz, I. 2021. “Modulating the Gut Microbiota With Dietary Interventions to Protect Against Cardiometabolic Disease.” Nature Reviews. Cardiology 18, no. 5: 305. 33658636 10.1038/s41569-021-00537-0

[fsn371242-bib-0023] Foddha, H. , I. B. Jeddou , H. Saoud , et al. 2025. “Impact of CCL5 Gene Polymorphisms on Coronary Artery Disease Risk and Severity in the Context of Diabetes Mellitus.” Scientific Reports 15, no. 1: 21104. 40594750 10.1038/s41598-025-07785-x PMC12216114

[fsn371242-bib-0024] Guo, Q. , X. Hou , Q. Cui , et al. 2024. “Pectin Mediates the Mechanism of Host Blood Glucose Regulation Through Intestinal Flora.” Critical Reviews in Food Science and Nutrition 64, no. 19: 6714–6736. 36756885 10.1080/10408398.2023.2173719

[fsn371242-bib-0025] Guo, Z. , J. Pan , H. Zhu , and Z. Chen . 2022. “Metabolites of Gut Microbiota and Possible Implication in Development of Diabetes Mellitus.” Journal of Agricultural and Food Chemistry 70, no. 20: 5945–5960. 35549332 10.1021/acs.jafc.1c07851

[fsn371242-bib-0026] He, Y. L. , G. Foteinos , S. Neelakantham , et al. 2013. “Differential Effects of Vildagliptin and Glimepiride on Glucose Fluctuations in Patients With Type 2 Diabetes Mellitus Assessed Using Continuous Glucose Monitoring.” Diabetes, Obesity & Metabolism 15, no. 12: 1111–1119. 10.1111/dom.12146 23782529

[fsn371242-bib-0027] Henriques, F. L. , I. Buckle , and J. M. Forbes . 2025. “Type 1 Diabetes Mellitus Prevention: Present and Future.” Nature Reviews. Endocrinology 21, no. 10: 608–622. 10.1038/s41574-025-01128-6 40527975

[fsn371242-bib-0028] Huang, L. , Y. Pan , K. Zhou , H. Liu , and S. Zhong . 2023. “Correlation Between Glycemic Variability and Diabetic Complications: A Narrative Review.” International Journal of General Medicine 16: 3083–3094. 37496596 10.2147/IJGM.S418520 PMC10368016

[fsn371242-bib-0029] Jiang, Y. , X. Yang , Y. Lei , S. Li , X. Chen , and L. Jiang . 2025. “Bacillus Paralicheniformis LN33 Fermented Feed Improves Growth Performance in Cherry Valley Ducks by Enhancing Immune Function and Intestinal Barrier Integrity.” Frontiers in Veterinary Science 12: 1619287. 40771965 10.3389/fvets.2025.1619287 PMC12327398

[fsn371242-bib-0030] Jung, H. S. 2015. “Clinical Implications of Glucose Variability: Chronic Complications of Diabetes.” Endocrinology and Metabolism 30, no. 2: 167–174. 26194076 10.3803/EnM.2015.30.2.167 PMC4508260

[fsn371242-bib-0031] Klimontov, V. V. , O. V. Saik , and A. I. Korbut . 2021. “Glucose Variability: How Does It Work.” International Journal of Molecular Sciences 22, no. 15: 7783. 34360550 10.3390/ijms22157783 PMC8346105

[fsn371242-bib-0032] Kommu, S. , P. P. Sharma , and R. M. Gabor . 2025. “Efficacy and Safety of Tirzepatide on Weight Loss in Patients Without Diabetes Mellitus: A Systematic Review and Meta‐Analysis of Randomized Controlled Trials.” Obesity Reviews 26, no. 11: e13961. 40510020 10.1111/obr.13961 PMC12531717

[fsn371242-bib-0033] Kovatchev, B. P. 2012. “Diabetes Technology: Markers, Monitoring, Assessment, and Control of Blood Glucose Fluctuations in Diabetes.” Scientifica 2012: 283821. 24278682 10.6064/2012/283821 PMC3820631

[fsn371242-bib-0034] Kraithong, S. , Y. Liu , S. Suwanangul , P. Sangsawad , A. Theppawong , and N. Bunyameen . 2025. “A Comprehensive Review of the Impact of Anthocyanins From Purple/Black Rice on Starch and Protein Digestibility, Gut Microbiota Modulation, and Their Applications in Food Products.” Food Chemistry 473: 143007. 39874887 10.1016/j.foodchem.2025.143007

[fsn371242-bib-0035] Kuenen, J. C. , R. Borg , D. J. Kuik , et al. 2011. “Does Glucose Variability Influence the Relationship Between Mean Plasma Glucose and HbA1c Levels in Type 1 and Type 2 Diabetic Patients.” Diabetes Care 34, no. 8: 1843–1847. 21700921 10.2337/dc10-2217 PMC3142023

[fsn371242-bib-0036] Li, Q. , Z. Gao , H. Wang , et al. 2018. “Intestinal Immunomodulatory Cells (T Lymphocytes): A Bridge Between Gut Microbiota and Diabetes.” Mediators of Inflammation 2018: 9830939. 29713241 10.1155/2018/9830939 PMC5866888

[fsn371242-bib-0037] Li, W. , Y. Wang , and G. Zhong . 2023. “Glycemic Variability and the Risk of Atrial Fibrillation: A Meta‐Analysis.” Frontiers in Endocrinology 14: 1126581. 37274320 10.3389/fendo.2023.1126581 PMC10232736

[fsn371242-bib-0038] Liang, L. , X. Su , Y. Guan , B. Wu , X. Zhang , and X. Nian . 2024. “Correlation Between Intestinal Flora and GLP‐1 Receptor Agonist Dulaglutide in Type 2 Diabetes Mellitus Treatment‐A Preliminary Longitudinal Study.” iScience 27, no. 5: 109784. 38711446 10.1016/j.isci.2024.109784 PMC11070333

[fsn371242-bib-0039] Lin, S. , S. Wang , P. Wang , et al. 2023. “Bile Acids and Their Receptors in Regulation of Gut Health and Diseases.” Progress in Lipid Research 89: 101210. 36577494 10.1016/j.plipres.2022.101210

[fsn371242-bib-0040] Lin, Y. , C. Lin , Y. Huang , et al. 2022. “Regimen Comprising GLP‐1 Receptor Agonist and Basal Insulin Can Decrease the Effect of Food on Glycemic Variability Compared to a Pre‐Mixed Insulin Regimen.” European Journal of Medical Research 27, no. 1: 273. 36463197 10.1186/s40001-022-00892-9 PMC9719195

[fsn371242-bib-0041] Liu, D. , Y. Zhang , Q. Wu , et al. 2024. “Exercise‐Induced Improvement of Glycemic Fluctuation and Its Relationship With Fat and Muscle Distribution in Type 2 Diabetes.” Journal of Diabetes 16, no. 4: e13549. 38584275 10.1111/1753-0407.13549 PMC10999499

[fsn371242-bib-0042] Liu, P. , M. Jin , P. Hu , et al. 2024. “Gut Microbiota and Bile Acids: Metabolic Interactions and Impacts on Diabetic Kidney Disease.” Current Research in Microbial Sciences 7: 100315. 39726973 10.1016/j.crmicr.2024.100315 PMC11670419

[fsn371242-bib-0043] Lu, J. , X. Ma , L. Zhang , et al. 2019. “Glycemic Variability Assessed by Continuous Glucose Monitoring and the Risk of Diabetic Retinopathy in Latent Autoimmune Diabetes of the Adult and Type 2 Diabetes.” Journal of Diabetes Investigation 10, no. 3: 753–759. 30306722 10.1111/jdi.12957 PMC6497773

[fsn371242-bib-0044] Lucci, C. , A. Marongiu , S. Genovese , M. Mazza , N. Cosentino , and G. Marenzi . 2025. “Hypoglycemia and Glycemic Variability in Acute Myocardial Infarction: The Lesser‐Known Aspects of Glycemic Control.” Cardiovascular Diabetology 24, no. 1: 309. 40739559 10.1186/s12933-025-02862-x PMC12312239

[fsn371242-bib-0045] Lundgrin, E. L. , and B. Hatipoglu . 2025. “Trending Modalities in Type 2 Diabetes Prevention.” Journal of Clinical Endocrinology and Metabolism 110, no. Supplement_2: S187–S192. 39998920 10.1210/clinem/dgaf040

[fsn371242-bib-0046] Lytrivi, M. , Y. Tong , E. Virgilio , X. Yi , and M. Cnop . 2025. “Diabetes Mellitus and the Key Role of Endoplasmic Reticulum Stress in Pancreatic β Cells.” Nature Reviews. Endocrinology 21, no. 9: 546–563. 10.1038/s41574-025-01129-5 40467970

[fsn371242-bib-0047] Ma, Q. , Y. Li , P. Li , et al. 2019. “Research Progress in the Relationship Between Type 2 Diabetes Mellitus and Intestinal Flora.” Biomedicine & Pharmacotherapy 117: 109138. 31247468 10.1016/j.biopha.2019.109138

[fsn371242-bib-0048] Ma, Y. , W. Wang , M. He , et al. 2024. “PVA‐Based Bulk Microneedles Capable of High Insulin Loading and pH‐Triggered Degradation for Multi‐Responsive and Sustained Hypoglycemic Therapy.” Biomaterials Science 12, no. 2: 507–517. 38088652 10.1039/d3bm01760e

[fsn371242-bib-0049] Marcovecchio, M. L. , M. Lucantoni , and F. Chiarelli . 2011. “Role of Chronic and Acute Hyperglycemia in the Development of Diabetes Complications.” Diabetes Technology & Therapeutics 13, no. 3: 389–394. 21299400 10.1089/dia.2010.0146

[fsn371242-bib-0050] Miao, H. , S. Zhang , X. Wu , P. Li , and Y. Zhao . 2025. “Tryptophan Metabolism as a Target in Gut Microbiota, Ageing and Kidney Disease.” International Journal of Biological Sciences 21, no. 10: 4374–4387. 40765836 10.7150/ijbs.115359 PMC12320236

[fsn371242-bib-0051] Monnier, L. , C. Colette , E. Renard , et al. 2025. “Glycemic Variability and Iatrogenic Hypoglycemia: How to Resolve.” Diabetes Research and Clinical Practice 226: 112360. 40614782 10.1016/j.diabres.2025.112360

[fsn371242-bib-0052] Moradi Baniasadi, M. , P. Arzhang , A. Setayesh , M. Moradi , E. Nasli‐Esfahani , and L. Azadbakht . 2025. “The Effect of Turmeric/Curcumin Supplementation on Anthropometric Indices in Subjects With Prediabetes and Type 2 Diabetes Mellitus: A GRADE‐Assessed Systematic Review and Dose‐Response Meta‐Analysis of Randomized Controlled Trials.” Nutrition & Diabetes 15, no. 1: 34. 40813857 10.1038/s41387-025-00386-7 PMC12354764

[fsn371242-bib-0053] Nyambuya, T. M. , B. B. Nkambule , S. E. Mazibuko‐Mbeje , et al. 2020. “A Meta‐Analysis of the Impact of Resveratrol Supplementation on Markers of Renal Function and Blood Pressure in Type 2 Diabetic Patients on Hypoglycemic Therapy.” Molecules 25, no. 23: 5645. 33266114 10.3390/molecules25235645 PMC7730696

[fsn371242-bib-0054] Ortega, H. I. , M. S. Udler , A. L. Gloyn , and S. A. Sharp . 2025. “Diabetes Mellitus Polygenic Risk Scores: Heterogeneity and Clinical Translation.” Nature Reviews. Endocrinology 21, no. 9: 530–545. 10.1038/s41574-025-01132-w PMC12614124 40467969

[fsn371242-bib-0055] Papachristoforou, E. , V. Lambadiari , E. Maratou , and K. Makrilakis . 2020. “Association of Glycemic Indices (Hyperglycemia, Glucose Variability, and Hypoglycemia) With Oxidative Stress and Diabetic Complications.” Journal Diabetes Research 2020: 7489795. 10.1155/2020/7489795 PMC7585656 33123598

[fsn371242-bib-0056] Park, J. , J. Kim , J. H. Noh , et al. 2022. “Pharmacokinetics of a Fixed‐Dose Combination Product of Dapagliflozin and Linagliptin and Its Comparison With co‐Administration of Individual Tablets in Healthy Humans.” Pharmaceutics 14, no. 3: 591. 35335967 10.3390/pharmaceutics14030591 PMC8952231

[fsn371242-bib-0057] Peng, W. , J. Huang , J. Yang , et al. 2019. “Integrated 16S rRNA Sequencing, Metagenomics, and Metabolomics to Characterize Gut Microbial Composition, Function, and Fecal Metabolic Phenotype in Non‐Obese Type 2 Diabetic Goto‐Kakizaki Rats.” Frontiers in Microbiology 10: 3141. 32038574 10.3389/fmicb.2019.03141 PMC6984327

[fsn371242-bib-0058] Pitsillides, A. N. , S. M. Anderson , and B. Kovatchev . 2011. “Hypoglycemia Risk and Glucose Variability Indices Derived From Routine Self‐Monitoring of Blood Glucose Are Related to Laboratory Measures of Insulin Sensitivity and Epinephrine Counterregulation.” Diabetes Technology & Therapeutics 13, no. 1: 11–17. 21175266 10.1089/dia.2010.0103 PMC3025766

[fsn371242-bib-0059] Qi, Y. , X. Zheng , L. Bi , et al. 2025. “Effects of Postprandial Exercise Timing on Blood Glucose and Fluctuations in Patients With Type 2 Diabetes Mellitus.” Journal of Sports Medicine and Physical Fitness 65, no. 1: 125–131. 39320030 10.23736/S0022-4707.24.16076-8

[fsn371242-bib-0060] Qin, L. , B. Fan , Y. Zhou , et al. 2025. “Targeted Gut Microbiome Therapy: Applications and Prospects of Probiotics, Fecal Microbiota Transplantation and Natural Products in the Management of Type 2 Diabetes.” Pharmacological Research 213: 107625. 39875017 10.1016/j.phrs.2025.107625

[fsn371242-bib-0061] Qin, W. , S. Zheng , L. Zhou , et al. 2025. “High‐Coverage Metabolomics Reveals Gut Microbiota‐Related Metabolic Traits of Type‐2 Diabetes in Serum.” Journal of Proteome Research 24, no. 4: 1649–1661. 40130449 10.1021/acs.jproteome.4c00507

[fsn371242-bib-0062] Radić, J. , A. Belančić , H. Đogaš , et al. 2025. “The Power of Movement: Linking Physical Activity With Nutritional Health and Blood Sugar Balance in a Dalmatian Type 2 Diabetic Population.” Nutrients 17, no. 1: 187. 39796621 10.3390/nu17010187 PMC11722635

[fsn371242-bib-0063] Sakai, C. , S. Tamaru , K. Sugai , H. Takeuchi , and R. Suzuki . 2025. “Clinical Use and Monitoring of Adverse Effects of Sodium‐Glucose Cotransporter‐2 Inhibitors in Persons With Type 1 Diabetes Mellitus.” Journal of Diabetes Investigation 16, no. 8: 1420–1429. 40418519 10.1111/jdi.70085 PMC12315242

[fsn371242-bib-0064] Santos, V. K. J. D. , M. L. De Lima Prado , G. S. N. D. Silva , et al. 2025. “Technosphere Insulin in the Treatment of Type 1 Diabetes Mellitus: A Systematic Review and Meta‐Analysis.” Journal of Diabetes and its Complications 39, no. 9: 109108. 40499400 10.1016/j.jdiacomp.2025.109108

[fsn371242-bib-0065] Sedighi, A. , T. Kou , H. Huang , and Y. Li . 2025. “Noninvasive on‐Skin Biosensors for Monitoring Diabetes Mellitus.” Nano Letters 18, no. 1: 16. 10.1007/s40820-025-01843-9 PMC12314300 40742490

[fsn371242-bib-0067] Shuai, J. , S. He , Y. Wang , J. Zhen , D. Yu , and M. Lv . 2025. “Dysregulated Tryptophan Metabolism and Gut Microbiota in Chronic Hypoxia‐Induced Pulmonary Hypertension Rats.” Journal of Pharmaceutical and Biomedical Analysis 266: 117111. 40818305 10.1016/j.jpba.2025.117111

[fsn371242-bib-0068] Smith‐Palmer, J. , M. Brändle , R. Trevisan , M. O. Federici , S. Liabat , and W. Valentine . 2014. “Assessment of the Association Between Glycemic Variability and Diabetes‐Related Complications in Type 1 and Type 2 Diabetes.” Diabetes Research and Clinical Practice 105, no. 3: 273–284. 25023992 10.1016/j.diabres.2014.06.007

[fsn371242-bib-0069] Sohail, M. U. , A. Althani , H. Anwar , R. Rizzi , and H. E. Marei . 2017. “Role of the Gastrointestinal Tract Microbiome in the Pathophysiology of Diabetes Mellitus.” Journal of Diabetes Research 2017: 9631435. 29082264 10.1155/2017/9631435 PMC5634576

[fsn371242-bib-0070] Šoupal, J. , J. Škrha , M. Fajmon , E. Horová , M. Mráz , and M. Prázný . 2014. “Glycemic Variability Is Higher in Type 1 Diabetes Patients With Microvascular Complications Irrespective of Glycemic Control.” Diabetes Technology & Therapeutics 16, no. 4: 198–203. 24401008 10.1089/dia.2013.0205

[fsn371242-bib-0071] Spiljar, M. , D. Merkler , and M. Trajkovski . 2017. “The Immune System Bridges the Gut Microbiota With Systemic Energy Homeostasis: Focus on TLRs, Mucosal Barrier, and SCFAs.” Frontiers in Immunology 8: 1353. 29163467 10.3389/fimmu.2017.01353 PMC5670327

[fsn371242-bib-0072] Su, X. , Y. Chen , and X. Yuan . 2025. “Gut Microbiota Modulation of Dementia Related Complications.” Aging and Disease. 10.14336/AD.2025.0108 PMC12727054 40072371

[fsn371242-bib-0073] Suez, J. , N. Zmora , G. Zilberman‐Schapira , et al. 2018. “Post‐Antibiotic Gut Mucosal Microbiome Reconstitution Is Impaired by Probiotics and Improved by Autologous FMT.” Cell 174, no. 6: 1406–1423. 30193113 10.1016/j.cell.2018.08.047

[fsn371242-bib-0074] Sun, L. , C. Xie , G. Wang , et al. 2018. “Gut Microbiota and Intestinal FXR Mediate the Clinical Benefits of Metformin.” Nature Medicine 24, no. 12: 1919–1929. 10.1038/s41591-018-0222-4 PMC6479226 30397356

[fsn371242-bib-0075] Takeuchi, T. , Y. Nakanishi , and H. Ohno . 2024. “Microbial Metabolites and Gut Immunology.” Annual Review of Immunology 42, no. 1: 153–178. 10.1146/annurev-immunol-090222-102035 38941602

[fsn371242-bib-0076] Tan, J. K. , L. Macia , and C. R. Mackay . 2023. “Dietary Fiber and SCFAs in the Regulation of Mucosal Immunity.” Journal of Allergy and Clinical Immunology 151, no. 2: 361–370. 36543697 10.1016/j.jaci.2022.11.007

[fsn371242-bib-0078] Teixeira, A. M. , R. Tsukamoto , C. T. Lopes , and R. Silva . 2017. “Risk Factors for Unstable Blood Glucose Level: Integrative Review of the Risk Factors Related to the Nursing Diagnosis.” Revista Latino‐Americana de Enfermagem 25: e2893. 28591300 10.1590/1518-8345.1688.2893 PMC5479373

[fsn371242-bib-0079] Thiruvengadam, M. , U. Subramanian , B. Venkidasamy , et al. 2023. “Emerging Role of Nutritional Short‐Chain Fatty Acids (SCFAs) Against Cancer via Modulation of Hematopoiesis.” Critical Reviews in Food Science and Nutrition 63, no. 6: 827–844. 34319824 10.1080/10408398.2021.1954874

[fsn371242-bib-0080] Tilg, H. , and A. R. Moschen . 2014. “Microbiota and Diabetes: An Evolving Relationship.” Gut 63, no. 9: 1513–1521. 24833634 10.1136/gutjnl-2014-306928

[fsn371242-bib-0081] Tong, X. , T. Jiang , J. Yang , et al. 2025. “Continuous Glucose Monitoring (CGM) System Based on Protein Hydrogel Anti‐Biofouling Coating for Long‐Term Accurate and Point‐Of‐Care Glucose Monitoring.” Biosensors & Bioelectronics 277: 117307. 40014948 10.1016/j.bios.2025.117307

[fsn371242-bib-0082] Tran, M. , J. R. Huh , and A. S. Devlin . 2025. “The Role of Gut Microbial Metabolites in the T Cell Lifecycle.” Nature Immunology 26, no. 8: 1246–1257. 40691327 10.1038/s41590-025-02227-2 PMC13124069

[fsn371242-bib-0083] Udayappan, S. D. , A. V. Hartstra , G. M. Dallinga‐Thie , and M. Nieuwdorp . 2014. “Intestinal Microbiota and Faecal Transplantation as Treatment Modality for Insulin Resistance and Type 2 Diabetes Mellitus.” Clinical and Experimental Immunology 177, no. 1: 24–29. 24528224 10.1111/cei.12293 PMC4089151

[fsn371242-bib-0085] Umpierrez, G. E. , and B. P. Kovatchev . 2018. “Glycemic Variability: How to Measure and Its Clinical Implication for Type 2 Diabetes.” American Journal of the Medical Sciences 356, no. 6: 518–527. 30447705 10.1016/j.amjms.2018.09.010 PMC6709582

[fsn371242-bib-0086] van Olden, C. , A. K. Groen , and M. Nieuwdorp . 2015. “Role of Intestinal Microbiome in Lipid and Glucose Metabolism in Diabetes Mellitus.” Clinical Therapeutics 37, no. 6: 1172–1177. 25922340 10.1016/j.clinthera.2015.03.008

[fsn371242-bib-0087] Venegas, D. P. , M. K. De la Fuente , G. Landskron , et al. 2019. “Short Chain Fatty Acids (SCFAs)‐Mediated Gut Epithelial and Immune Regulation and Its Relevance for Inflammatory Bowel Diseases.” Frontiers in Immunology 10: 277. 30915065 10.3389/fimmu.2019.00277 PMC6421268

[fsn371242-bib-0088] Verma, S. , A. K. Mishra , A. Mishra , K. Y. Thajudeen , H. Singh , and G. Khan . 2025. “A Systematic Review on Effect of Bifidobacterium Isolated From Skin Microbiota on GLP‐1 Production to Alleviate Human Ailments.” Probiotics and Antimicrobial Proteins. 10.1007/s12602-025-10709-w 40768022

[fsn371242-bib-0089] Wang, G. , Y. Yu , Y. Wang , et al. 2019. “Role of SCFAs in Gut Microbiome and Glycolysis for Colorectal Cancer Therapy.” Journal of Cellular Physiology 234, no. 10: 17023–17049. 30888065 10.1002/jcp.28436

[fsn371242-bib-0090] Wang, H. , C. Tang , Z. Gao , et al. 2021. “Potential Role of Natural Plant Medicine *Cyclocarya paliurus* in the Treatment of Type 2 Diabetes Mellitus.” Journal of Diabetes Research 2021: 1655336. 34988228 10.1155/2021/1655336 PMC8723876

[fsn371242-bib-0091] Wen, Y. , Z. Sun , S. Xie , et al. 2022. “Intestinal Flora Derived Metabolites Affect the Occurrence and Development of Cardiovascular Disease.” Journal of Multidisciplinary Healthcare 15: 2591–2603. 36388628 10.2147/JMDH.S367591 PMC9656419

[fsn371242-bib-0092] Wu, W. , Q. Chai , and Z. Zhang . 2021. “Glucose Fluctuation Accelerates Cardiac Injury of Diabetic Mice via Sodium‐Dependent Glucose Cotransporter 1 (SGLT1).” Archives of Biochemistry and Biophysics 709: 108968. 34153296 10.1016/j.abb.2021.108968

[fsn371242-bib-0093] Xu, J. , N. Wang , L. Yang , J. Zhong , and M. Chen . 2024. “Intestinal Flora and Bile Acid Interactions Impact the Progression of Diabetic Kidney Disease.” Frontiers in Endocrinology 15: 1441415. 39371929 10.3389/fendo.2024.1441415 PMC11449830

[fsn371242-bib-0094] Yan, R. , L. Zhang , Y. Chen , Y. Zheng , P. Xu , and Z. Xu . 2025. “Therapeutic Potential of Gut Microbiota Modulation in Epilepsy: A Focus on Short‐Chain Fatty Acids.” Neurobiology of Disease 209: 106880. 40118219 10.1016/j.nbd.2025.106880

[fsn371242-bib-0095] Yin, C. , X. Wen , G. Dang , et al. 2024. “Modulation of Pectin on Intestinal Barrier Function via Changes in Microbial Functional Potential and Bile Acid Metabolism.” Journal of Nutritional Biochemistry 124: 109491. 37865382 10.1016/j.jnutbio.2023.109491

[fsn371242-bib-0096] Yoo, S. , S. O. Chin , S. A. Lee , and G. Koh . 2015. “Factors Associated With Glycemic Variability in Patients With Type 2 Diabetes: Focus on Oral Hypoglycemic Agents and Cardiovascular Risk Factors.” Endocrinology and Metabolism 30, no. 3: 352–360. 26248860 10.3803/EnM.2015.30.3.352 PMC4595361

[fsn371242-bib-0097] Zhang, J. , Y. Sun , L. Zhao , et al. 2019. “SCFAs‐Induced GLP‐1 Secretion Links the Regulation of Gut Microbiome on Hepatic Lipogenesis in Chickens.” Frontiers in Microbiology 10: 2176. 31616396 10.3389/fmicb.2019.02176 PMC6775471

[fsn371242-bib-0098] Zhang, L. , X. Wang , and X. Zhang . 2022. “Modulation of Intestinal Flora by Dietary Polysaccharides: A Novel Approach for the Treatment and Prevention of Metabolic Disorders.” Food 11, no. 19: 2961. 10.3390/foods11192961 PMC9562892 36230037

[fsn371242-bib-0099] Zhang, Y. , Y. Han , Q. Zheng , and X. Lin . 2025. “Commentary on ‘Liver Function Effects of SGLT2 Inhibitor and GLP‐1 Receptor Agonist Combination Treatment in Patients With Type 2 Diabetes (Post Hoc Analysis of RECAP Study)’.” Journal of Diabetes Investigation 16: 2120–2122. 40827446 10.1111/jdi.70143 PMC12578661

[fsn371242-bib-0100] Zhang, Z. , N. Wang , L. Qian , et al. 2020. “Glucose Fluctuations Promote Aortic Fibrosis Through the ROS/p38 MAPK/Runx2 Signaling Pathway.” Journal of Vascular Research 57, no. 1: 24–33. 31715615 10.1159/000503608

[fsn371242-bib-0101] Zhao, P. , Y. Chen , S. Zhou , and F. Li . 2025. “Microbial Modulation of Tryptophan Metabolism Links Gut Microbiota to Disease and Its Treatment.” Pharmacological Research 219: 107896. 40763909 10.1016/j.phrs.2025.107896

[fsn371242-bib-0102] Zheng, J. , J. Chen , W. Zhang , et al. 2025. “Tryptophan Attenuates Acute Hypoxic Stress‐Induced Intestinal Injury Through the Modulation of Intestinal Barrier Integrity and Gut Microbiota Homeostasis.” Genes & Diseases 12, no. 6: 101627. 40727587 10.1016/j.gendis.2025.101627 PMC12301910

[fsn371242-bib-0103] Zhou, T. , Z. Jin , R. Jiang , and W. Li . 2025. “Gut Microbiota Modulation by Traditional Chinese Medicine: A Translational Strategy for Metabolic Dysfunction‐Associated Steatotic Liver Disease.” Frontiers in Pharmacology 16: 1600439. 40556760 10.3389/fphar.2025.1600439 PMC12185430

[fsn371242-bib-0104] Zikou, E. , C. Koliaki , and K. Makrilakis . 2024. “The Role of Fecal Microbiota Transplantation (FMT) in the Management of Metabolic Diseases in Humans: A Narrative Review.” Biomedicine 12, no. 8: 1871. 10.3390/biomedicines12081871 PMC11352194 39200335

[fsn371242-bib-0105] Zong, W. , E. S. Friedman , S. R. Allu , et al. 2024. “Disruption of Intestinal Oxygen Balance in Acute Colitis Alters the Gut Microbiome.” Gut Microbes 16, no. 1: 2361493. 38958039 10.1080/19490976.2024.2361493 PMC11225921

